# Composite lymphoma associating diffuse large B‐cell lymphoma with classical Hodgkin lymphoma

**DOI:** 10.1002/jha2.590

**Published:** 2022-10-06

**Authors:** Maïlis Lauwers, Valéry Daubie, Hussein Farhat, Laureen Rocq, Anne‐Laure Trépant, Laura Vanoverschelde

**Affiliations:** ^1^ Department of Haematology Laboratoire Hospitalier Universitaire de Bruxelles – Universitair Laboratorium Brussel (LHUB‐ULB) Université Libre de Bruxelles (ULB) Brussels Belgium; ^2^ Lebanese American University Medical Center Rizk Hospital Beirut Lebanon; ^3^ Department of Pathology Erasme University Hospital Université Libre de Bruxelles (ULB) Brussels Belgium

**Keywords:** classical Hodgkin lymphoma, composite lymphoma, diffuse large B‐cell lymphoma

**Abbreviations**
CHL: classical Hodgkin lymphoma
CL: composite lymphoma
CLA: common leukocyte antigen
DLBCL: diffuse large B‐cell lymphoma

1

A 55‐year‐old man with a 1‐month history of asthenia, right hypochondrial pain, and weight loss was referred to the emergency department for hepatic mass evaluation. Besides hypertension, he had no relevant pre‐existing conditions.

Physical examination revealed a palpable right inguinal lymph node. Complete blood count was normal apart from mild leukocytosis (15.2 × 10^9^/l, N: 3.5–10.0 × 10^9^/l) with neutrophilia. Liver function tests were altered (gamma‐glutamyl transferase: 134 U/l, N: 8–61 U/l; alkaline phosphatase: 167 U/l; N: 40–129 U/l). C‐reactive protein level was elevated at 104 mg/l (N: <5 mg/l). Lactate dehydrogenase was within normal ranges. Abdominal computed tomography scan revealed a large necrotic mass in the liver, a second necrotic mass adjacent to the superior mesenteric vessels, and numerous subdiaphragmatic lymph nodes (aorta, right kidney, retro‐crural, mesenteric, iliac, and inguinal). Hypermetabolic activity was confirmed by positron emission tomography.

A right inguinal lymph node biopsy was performed. Histological examination revealed infiltration by sheets of large lymphoid cells displaying irregularly shaped nuclei with macronucleoli and moderately abundant, finely granular cytoplasm (Figure 1C). Immunohistochemistry showed that these cells were positive for common leukocyte antigen (CLA), CD20, and CD30 and negative for anaplastic lymphoma kinase (Figure 1D‐F). The Ki‐67 proliferation index was high (∼90%). *BCL‐2, BCL‐6*, and *MYC* rearrangements were not detected by fluorescence in situ hybridization. Epstein‐Barr virus‐encoded small RNA was negative. A first diagnosis of diffuse large B‐cell lymphoma (DLBCL) was suggested.

**FIGURE 1:**
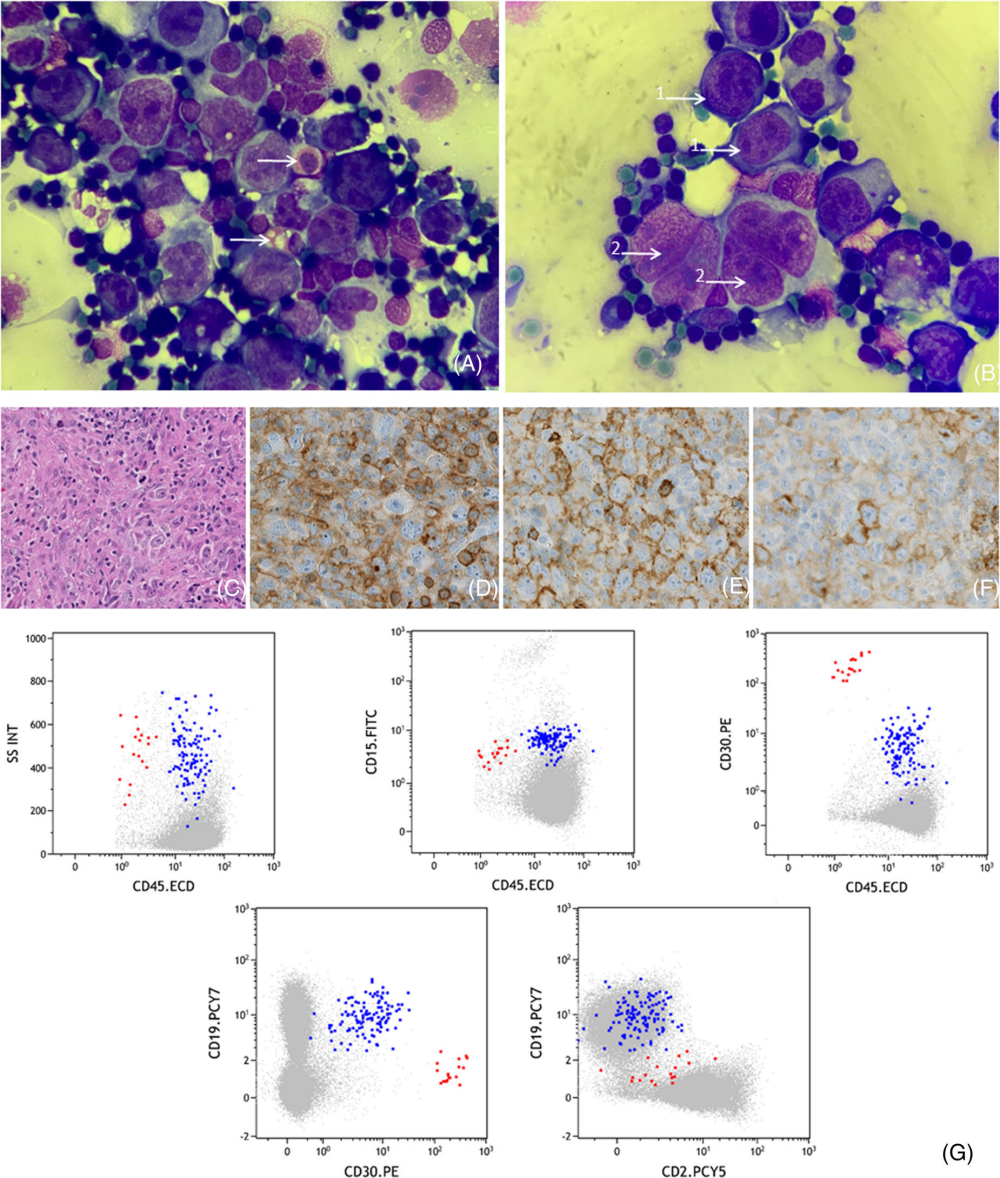
Inguinal lymph node biopsy imprint showing a heterogeneous background containing (A) eosinophils and (B, 1) large cells with various nucleocytoplasmic ratios, occasionally irregular nuclear contour, dispersed and delicately condensed mixed chromatin, multiple prominent nucleoli and deeply basophilic cytoplasm and (B, 2) scattered cells with sternbergoid features and decreased cytoplasmic basophilia (May Grunwald Giemsa, 1000x). Histology of the inguinal lymph node showing (C) sheets of large lymphoid cells displaying irregularly shaped nuclei with macronucleoli and moderately abundant, finely granular cytoplasm (hematoxylin and eosin, 200×). Immunohistochemical studies showing positivity for (D) CLA (200×), (E) CD20 (200×), and (F) CD30 (200×). (G) Flow cytometry charts showing the presence of two cell populations with different immunophenotypes: a population with high side scatter (SSC), CD19+, CD20+, CD22+, CD15+dim, CD30+dim, CD45+ cells (blue population) and a few CD19‐, CD20‐, CD15+dim, CD30+bright, CD45‐ cells (red population).

Touch imprint cytology revealed areas of large cells with various nucleocytoplasmic ratios, occasionally irregular nuclear contour, dispersed and delicately condensed mixed chromatin, multiple prominent nucleoli and deeply basophilic cytoplasm, consistent with a DLBCL component. Additionally, scattered cells showing sternbergoid features with decreased cytoplasmic basophilia in a heterogeneous background containing eosinophils were identified (Figure 1A‐B). Molecular analysis revealed a monoclonal immunoglobulin gene rearrangement confirming the diagnosis of the B‐cell component while morphological and flow cytometric findings highlighted the features of the Hodgkin component of the lymphoma. Indeed, in addition to a predominant polyclonal B‐cell population, flow cytometric analysis (Figure 1G) revealed the presence of two cell populations with different immunophenotypes: a population of high side scatter (SSC), CD19+, CD20+, CD22+, CD15+dim, CD30+dim, CD45+ cells (blue population) and a few CD19‐, CD20‐, CD15+dim, CD30+bright, CD45‐ cells (red population). Findings raised a differential diagnosis between unclassifiable B‐cell lymphoma with characteristics intermediate between DLBCL and classical Hodgkin lymphoma (CHL) versus composite lymphoma (CL) associating DLBCL with CHL. Based on the CD30 expression intensity as well as the negativity for CD45 and CD19, the final diagnosis of CL associating DLBCL with CHL was retained. After a multidisciplinary discussion, a treatment plan of rituximab, ifosfamide, carboplatin, etoposide regimen, dexamethasone, plus high dose radiation therapy was initiated.

CL is defined by the concomitant occurrence of two or more distinct lymphoma types in the same organ or anatomical site [1]. The incidence of this entity is low, varying from 1% to 4.7% of all newly diagnosed lymphomas per year [2]. CL with CHL and DLBCL is even more infrequent [3]. Less than 30 cases have been reported in the literature [4, 5]. The ratio of men to women is estimated at about 1.4:1 with a mean age of 51 years [5]. The lymph nodes were the most frequently affected sites (64%), and only 10% of patients have mesenteric involvement [4]. The association of Epstein‐Barr virus and CLs with CHL and DLBCL was observed in about 31% of the cases and was found to be associated with a worsened survival (average survival time 3 vs. 15 months) [5]. The etiology is not clear and is believed to be closely related to the deficiency of immune function and the deregulation of cell signaling pathways (e.g., NF‐KB) [6]. No optimal treatment is clearly defined so far mainly due to the lack of systematic evaluation of therapies. Treatment should target the most aggressive component of the lymphoma or the identified and targetable mutations [7].

This case of composite DLBCL and CHL illustrates the importance of integrating cytological, immunohistochemical, immunophenotyping, cytogenetic, and molecular studies in the diagnosis of such rare cases.

## AUTHOR CONTRIBUTIONS

M.L., V.D., H.F. et L.V. collected the data for the manuscript. M.L. and V.D. participated in the writing of the paper. L.R. and A‐L.T. performed the immunohistochemical analysis and reviewed the manuscript. L.V. and H.F. were involved in critical revision of the manuscript. All authors approved submission of the manuscript.

## FUNDING INFORMATION

The authors received no specific funding for this work.

## CONFLICT OF INTEREST

The authors declare no conflict of interest.

## ETHICS STATEMENT

No informed consent was required.

## Data Availability

Data availability can be obtained upon request to the corresponding author.

## References

[1] Kim H , Hendrickson R , Dorfman RF . Composite lymphoma. Cancer. 1977;40(3):959–76. 332325 10.1002/1097-0142(197709)40:3<959::aid-cncr2820400302>3.0.co;2-3

[2] National Cancer Institute sponsored study of classifications of non‐Hodgkin's lymphomas: summary and description of a working formulation for clinical usage. The Non‐Hodgkin's Lymphoma Pathologic Classification Project. Cancer. 1982;49(10):2112–35. 6896167 10.1002/1097-0142(19820515)49:10<2112::aid-cncr2820491024>3.0.co;2-2

[3] Mokhtar NM . Review article composite lymphoma. J Egypt Natl Canc Inst. 2007;19(3):171–5. 19190689

[4] Wang J , Zhang R . Composite lymphoma of cervical lymph nodes with classical Hodgkin lymphoma and diffuse large B‐cell lymphoma: a case report and literature review. Ann Palliat Med. 2020;9(5):3651–62. 32921109 10.21037/apm-20-1290

[5] Goyal G , Nguyen AH , Kendric K , Caponetti GC . Composite lymphoma with diffuse large B‐cell lymphoma and classical Hodgkin lymphoma components: a case report and review of the literature. Pathol Res Pract. 2016;212(12):1179–90. 27887763 10.1016/j.prp.2016.11.002

[6] Küppers R . Molecular biology of Hodgkin lymphoma. Hematology. 2009;9(1):491–6. 10.1182/asheducation-2009.1.491 20008234

[7] Küppers R , Dührsen U , Hansmann M‐L . Pathogenesis, diagnosis, and treatment of composite lymphomas. Lancet Oncol. 2014;15(10):435–46. 10.1016/S1470-2045(14)70153-6 25186047

